# Supplementary material for: Mechanically Strong, Liquid-Resistant Photothermal Bioplastic Constructed from Cellulose and Metal-Organic Framework for Light-Driven Mechanical Motion
Source: Molecules. 2021 Jul 23;26(15):4449. doi: 10.3390/molecules26154449 (PMC8348137; doi:10.3390/molecules26154449)
Supplement: Supplementary file 1 [file molecules-26-04449-s001.zip › molecules-1277814-supplementary for XML.pdf]

molecules

MDPI

Supplementary Material

Mechanically Strong, Liquid-Resistant Photothermal Bioplastics Constructed from Cellulose and Metal-Organic Frameworks for Light-Driven Mechanical Motion

Lijian Sun, Limei Li, Xianhui An and Xueren Qian *

Key Laboratory of Bio-based Material Science & Technology, Northeast Forestry University, Ministry of Education, Harbin 150040, China; sunlijian0308@163.com (L.S.); limeili937@foxmail.com (L.L.); anxianh509@163.com (X.A.)

* Correspondence: qianxueren@edu.cn; Tel.: +86-133-0464-2918

Supplementary Informations

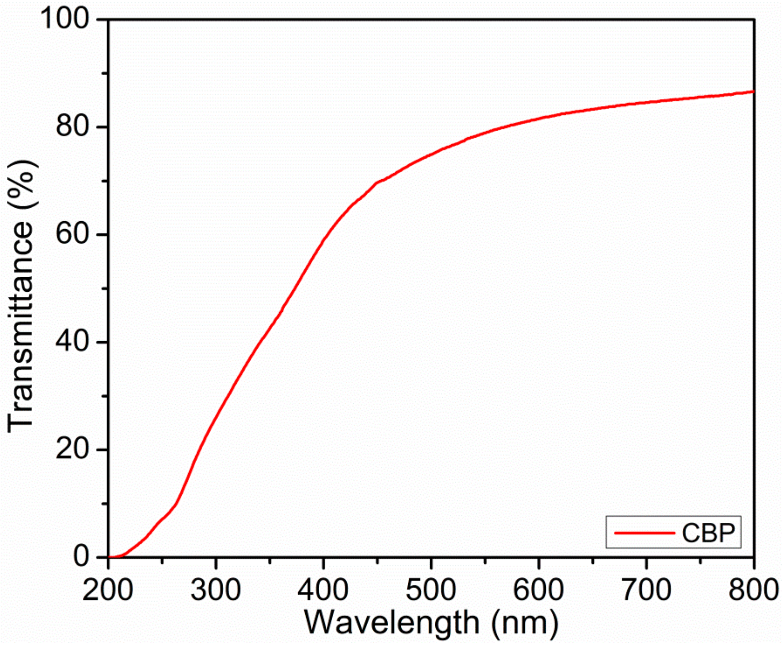

Figure S1. UV-vis spectrum of the CBP.

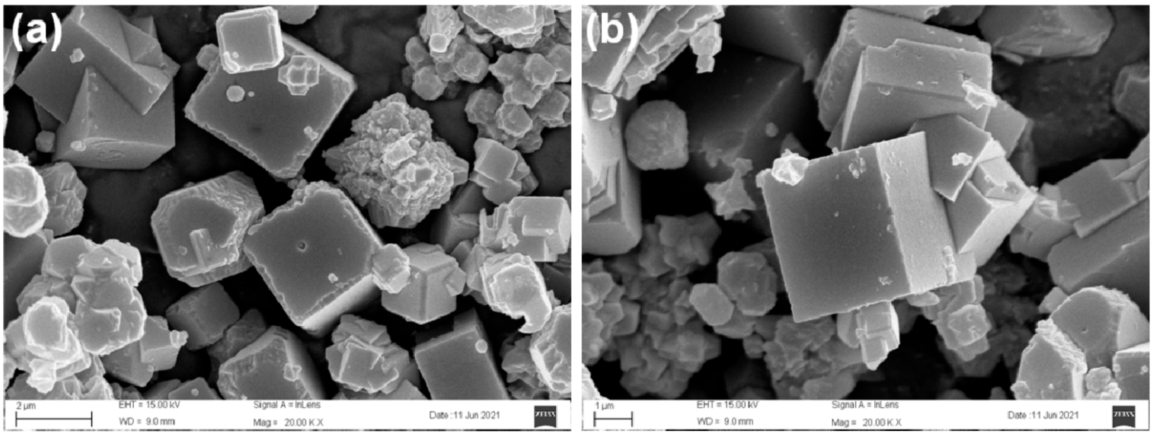

Figure S2. SEM images of PB.

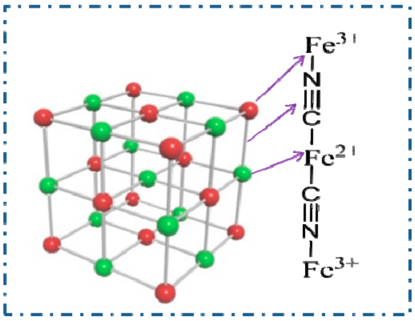

Figure S3. The crystal structure of PB.

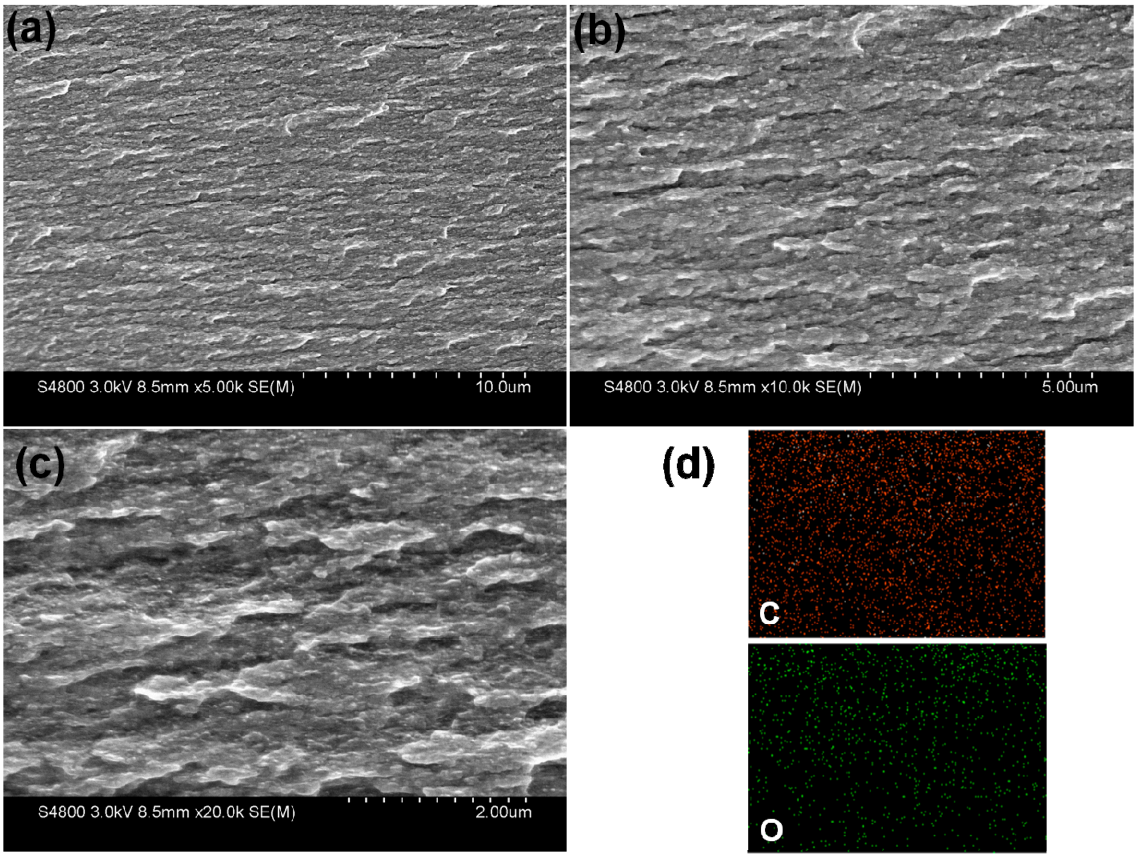

Figure S4. Cross-sectional SEM images of CBP.

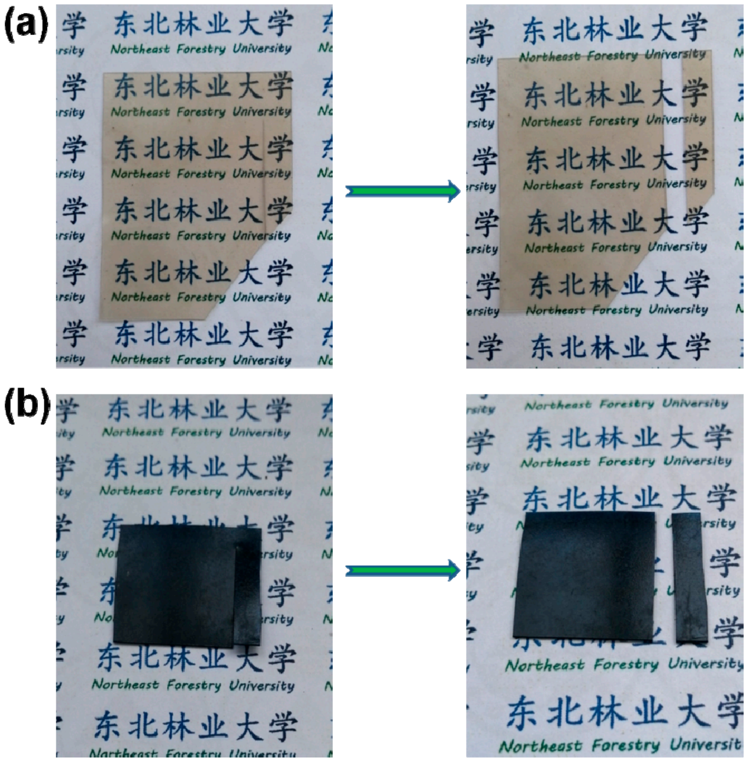

Figure S5. Tailoring process of CBP (**a**) and PCBP-2 (**b**).

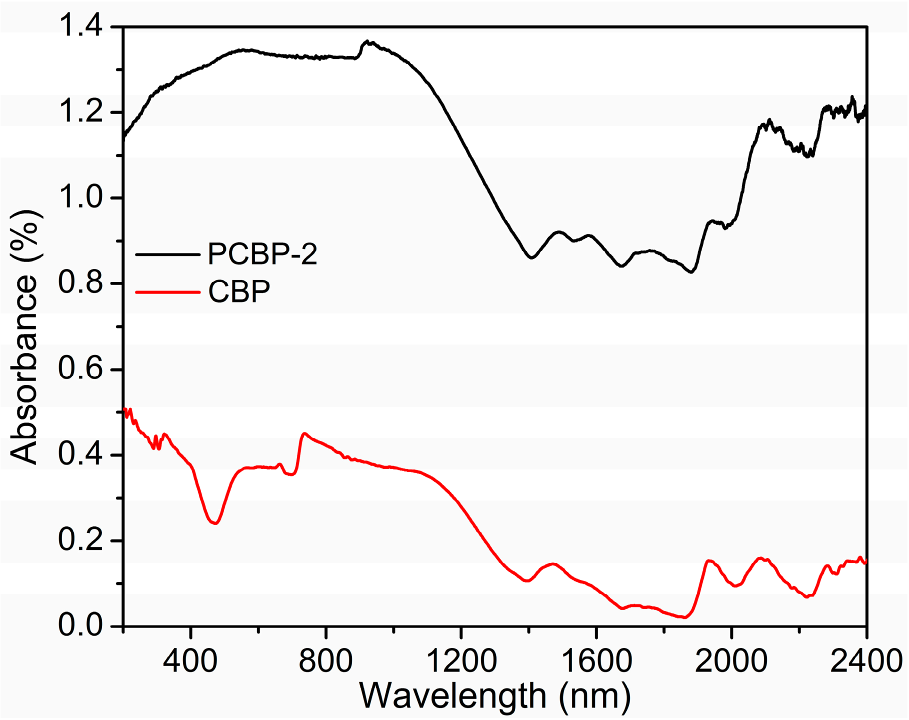

Figure S6. Absorption spectra of the pristine CBP and PCBP-2 in the wavelength range of 200–2400 nm.

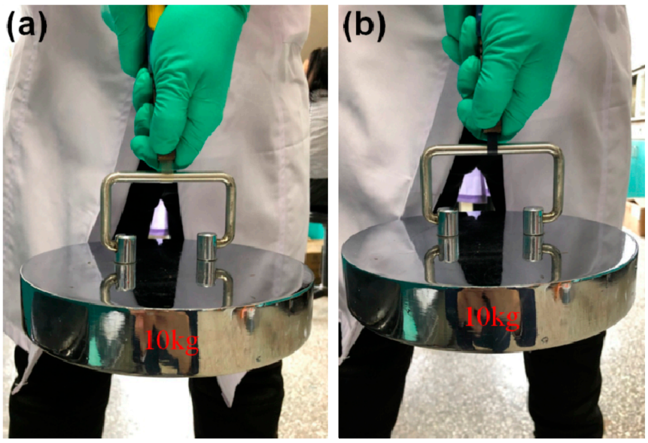

Figure S7. Digital photographs showing mechanical robustness of CBP (**a**) and PCBP-2 (**b**) in supporting a metal flattener (10 kg).
